# Hybrid Top-Down/Bottom-Up Fabrication of a Highly Uniform and Organized Faceted AlN Nanorod Scaffold

**DOI:** 10.3390/ma11071140

**Published:** 2018-07-05

**Authors:** Pierre-Marie Coulon, Gunnar Kusch, Philip Fletcher, Pierre Chausse, Robert W. Martin, Philip A. Shields

**Affiliations:** 1 Centre of Nanoscience & Nanotechnology & Department of Electronic and Electrical Engineering, University of Bath, Bath BA2 7AY, UK; P.J.Fletcher@bath.ac.uk (P.F.); P.J.P.Chausse@bath.ac.uk (P.C.); P.Shields@bath.ac.uk (P.A.S.); 2 Department of Physics, SUPA, University of Strathclyde, Glasgow G4 0NG, UK; gunnar.kusch@strath.ac.uk (G.K.); r.w.martin@strath.ac.uk (R.W.M.)

**Keywords:** AlN, nanorod, displacement Talbot lithography, etching, MOVPE, TEM, cathodoluminescence

## Abstract

As a route to the formation of regular arrays of AlN nanorods, in contrast to other III-V materials, the use of selective area growth via metal organic vapor phase epitaxy (MOVPE) has so far not been successful. Therefore, in this work we report the fabrication of a highly uniform and ordered AlN nanorod scaffold using an alternative hybrid top-down etching and bottom-up regrowth approach. The nanorods are created across a full 2-inch AlN template by combining Displacement Talbot Lithography and lift-off to create a Ni nanodot mask, followed by chlorine-based dry etching. Additional KOH-based wet etching is used to tune the morphology and the diameter of the nanorods. The resulting smooth and straight morphology of the nanorods after the two-step dry-wet etching process is used as a template to recover the AlN facets of the nanorods via MOVPE regrowth. The facet recovery is performed for various growth times to investigate the growth mechanism and the change in morphology of the AlN nanorods. Structural characterization highlights, first, an efficient dislocation filtering resulting from the ~130 nm diameter nanorods achieved after the two-step dry-wet etching process, and second, a dislocation bending induced by the AlN facet regrowth. A strong AlN near band edge emission is observed from the nanorods both before and after regrowth. The achievement of a highly uniform and organized faceted AlN nanorod scaffold having smooth and straight non-polar facets and improved structural and optical quality is a major stepping stone toward the fabrication of deep UV core-shell-based AlN or Al_x_Ga_1-x_N templates.

## 1. Introduction

Since their emergence in early 2000, deep-ultraviolet (DUV) aluminum gallium nitride (AlGaN)-based light emitting diodes (LEDs) have gained significant attention owing to their wide range of applications, e.g., UV curing [1], medical diagnostics, phototherapy [2], optical sensing [3,4], security, communications [5], sterilization, and water and air purification [6,7,8,9]. However, despite the effort made to increase the external quantum efficiency (EQE) of UV LEDs, the reported values are still low, and barely reach a few percent in the UVC wavelength range, which is too low for commercial applications [8,10]. Various technical issues limit this efficiency, such as high defect densities in AlN and Al_x_Ga_1−x_N materials [11,12,13,14], low carrier injection and related difficulties to efficiently p-dope Al_x_Ga_1−x_N alloys with high Al content [15,16], issues associated with the internal electric field in quantum wells [17,18], and poor light extraction [19,20,21,22].

Some of these challenges can be addressed with the use of 3D core-shell nanostructures owing to their low defect density [23,24,25], reduced quantum-confined Stark effect, high-quality non-polar growth, larger emitting surface [26,27], and improved extraction efficiency [28,29,30]. However, approaches to create AlN nanorod templates to act as a core for the subsequent growth of AlGaN/AlN MQW shells are immature compared to the fabrication of GaN nanorod templates [31,32,33]. Whilst there exist a large number of approaches, techniques, or strategies to create AlN nanorods, the vast majority report the formation of highly crystallographically-misoriented and inclined nanorods with respect to the substrate, with non-uniform diameters and poor position control [34,35]. These are critical parameters for the subsequent growth of active shell material and for fabricating devices. Indeed most, if not all, visible InGaN/GaN-based, electrically-injected LEDs rely on nanorods created through selective area growth, which results in good control of orientation, uniformity, and positioning [36,37]. However, in the case of AlN, the SAG of Al(Ga)N-based nanorods has not been achieved due to the very high sticking coefficient and the low diffusion length of Al atoms [38]. The alternative of growing nitrogen-polar Al(Ga)N nanorods on Si by molecular beam epitaxy (MBE) [39,40,41] has successfully shown the potential of AlN-based nanostructures to circumvent the challenges of planar technology. However, these nanorods are only suitable for the growth of heterostructures with an axial rather than a core-shell geometry, with only the latter being able to provide a larger active volume. In addition, a GaN pedestal grown on Si is systematically used to initiate the nucleation of AlN nanostructures [39,40,41,42,43], which is detrimental to any UVC emitting device due to reabsorption of the emitted light by the GaN pedestal and poor transparency of the Si substrate for this spectral region.

An alternative route for creating AlN nanorods involves combining top-down etching and MOVPE regrowth. Already demonstrated for InGaN/GaN core-shell nanorod arrays [44,45,46], this route allows the fabrication of uniform and organized AlN nanorod templates on a sapphire transparent substrate without any detrimental GaN present and the fabrication of subsequent AlGaN/AlN core-shell devices [47,48]. However, the transfer of technology from GaN to AlN materials to create high-quality nanorod arrays with vertical sidewalls and well-defined facets is not trivial due to the different chemistry associated with the Al versus the Ga species during both the etching and growth steps.

Therefore, in this paper we demonstrate and detail a process for the fabrication of highly organised and uniform AlN nanorod arrays with straight nonpolar facets that are suitable for subsequent active layer growth. We explain the success of the recipe for successfully obtaining AlN nanorod templates having straight and smooth *m*-plane sidewall facets, and compare our results with those previously reported. We present results from transmission electron microscopy and hyperspectral cathodoluminescence that show the high structural and optical properties of the AlN nanorods and confirm their suitability for use in the hybrid top-down/bottom-up approach to achieve non-polar core-shell AlGaN/AlN nanorod arrays on a wafer scale and higher efficiency deep-UV light emitting devices.

## 2. Materials and Methods

**Figure 1:**
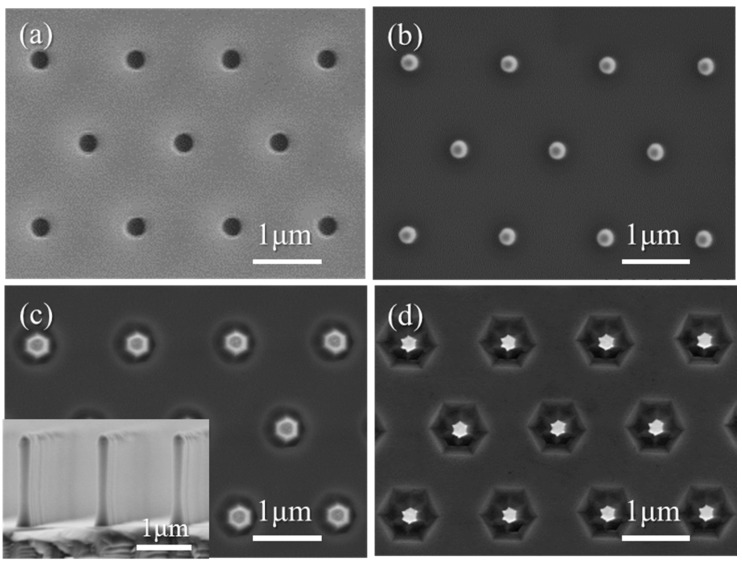
Secondary-electron plan-view SEM images of (**a**) the hole pattern in resist after DTL exposure and development, (**b**) the AlN template surface with the Au/Ni metal dot mask after lift-off, and (**c**) after Cl_2_/Ar plasma etching and (**d**) after AZ400k wet etching. The inset displays a cross-section image of the nanorod after Cl_2_/Ar plasma etching.

The AlN nanorod templates were fabricated by combining top-down etching and bottom-up MOVPE regrowth. The following process used a ~4–5 µm AlN template grown by MOVPE on a (0001) sapphire substrate sourced commercially (Nanowin, Suzhou, China). After spin-coating the wafer with a bottom antireflective layer (BARC) (Wide-30W, Brewer Science) and a high-contrast positive resist (Ultra-i 123, Dow Electronic Materials, Dongguan, China) Displacement Talbot lithography (DTL) (PhableR 100, Eulitha, Switzerland) was employed to expose the resist through an amplitude mask comprising holes with a diameter of about 800 nm in a hexagonal pattern with a 1.5 µm pitch. By optimizing the resist thickness, the exposure time, and the development time, ~260 nm diameter hole openings in the resist were created along with the formation of an undercut profile in the underlying bottom antireflective layer (Figure 1a). Metal layers (10 nm Au and 200 nm Ni) were then deposited via e-beam evaporation, and subsequent lift-off with MF-CD-26 developer (Dow Electronic Materials) was used to create a homogenous 1.5 µm pitch array of metal dots of diameter ~250 nm on the surface of a full 2-inch AlN template (Figure 1b).

Inductively coupled plasma (ICP) dry etching (System 100 Cobra, Oxford Instruments, Bristol, UK) was then used to obtain the AlN nanorods (inset in Figure 1c). A Cl_2_/Ar chemistry was used with flows of 50 sccm and 10 sccm, respectively, a temperature set to 150 °C, a pressure of 15 mTorr, 80 W RF power, and 800 W ICP source power. The AlN nanorods were then cleaned in buffered oxide etchant (BOE 5:1) to remove any passivation layer created after ICP etching. AZ400K potassium hydroxide (KOH)-based developer (AZ Electronic Materials, Wiesbaden, Germany) was then used at room temperature for 8 min to further smooth and shrink the AlN nanorod diameters (Figure 1d). Finally, the metal mask was etched away in aqua-regia solution (HCl:HNO_3_, 3:1). The bottom-up regrowth of AlN was carried out in a 1 × 2” horizontal MOVPE reactor (AIX 200/4HT RF-S, AIXTRON, Germany). The growth conditions employed to create smooth facets on the etched AlN nanorods were as follows: a growth temperature of 1100 °C, a pressure of 20 mbar, a TMAl flow rate of 10 sccm, an NH_3_ flow rate of 4000 sccm (V/III ratio of 30554), and H_2_ as the carrier gas. ICP etching and AlN regrowth conditions follow the optimum parameters presented in our previous work [48].

The morphology and structural characterization of AlN nanorods were investigated using a TEM at 200 kV (JEM-2100, JEOL, Tokyo, Japan). The nanorods used for TEM observations were mechanically removed from the wafer and deposited on a carbon grid.

Optical characterization was performed via cathodoluminescence hyperspectral imaging measurements in a modified field-emission SEM (Quanta 250, FEI, Hillsboro, OR, USA). Measurements were carried out at room temperature using electron energies of 15 keV and beam currents of approximately 7 nA. A 125 mm focal length spectrograph with a 600 lines/mm grating and 50 μm entrance slit was used, coupled to a cooled electron-multiplying charge-coupled device (EMCCD) detector [49].

## 3. Results and Discussion

### 3.1. Morphology and Structural Quality of Top-Down Etched AlN Nanorods

**Figure 2:**
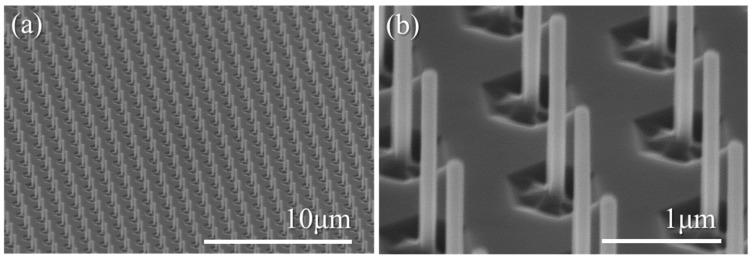
(**a**) Low and (**b**) high magnification, tilted, secondary-electron SEM images of the AlN nanorod arrays after the two-step dry-wet etching process and metal mask removal.

Figure 2 shows tilted SEM images of an AlN nanorod array after the two step dry-wet etching process. The low magnification SEM image (Figure 2a) provides an example of the high organization and uniformity of the AlN etched nanorods across a full 2-inch wafer. The higher magnification image (Figure 2b) highlights the verticality of the sidewalls of the AlN nanorods, with the latter having an average diameter of ~130 nm and a height of 1.8 µm. Compared to a single ICP-based dry etch step, the addition of a KOH-based wet etch step improves the morphology of the nanorods and further reduces their diameters, as observed between Figure 1c,d. Note, that the high physical ion bombardment during ICP etching induces the formation of circular trenches at the base of the nanorods, which then become larger hexagonal trenches due to the preferential KOH etching along the $10\bar{1}0$ direction.

**Figure 3:**
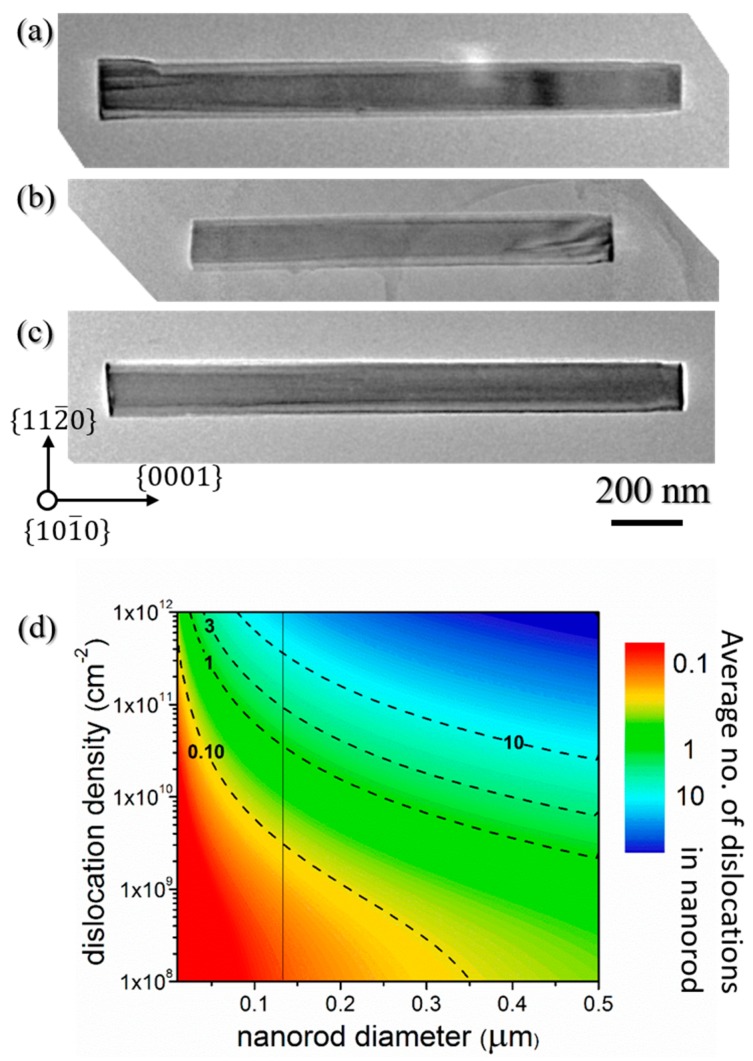
(**a**–**c**) TEM pictures of three different AlN etched nanorods acquired along the [$10\bar{1}0$] zone axis. The left and right hand-side of each image are related to the bottom and top of the nanorod, respectively. Note, the nanorod in (**b**) has been broken during mechanical removal and hence is shorter; (**d**) average number of dislocations from the template which remain in the nanorod, calculated for different etched nanorod diameters as a function of the dislocation density in the planar template. The dashed lines are contour lines to guide the eye. The vertical straight line corresponds to a nanorod diameter of 130 nm.

The morphology and structural quality of the etched nanorods was further analyzed by transmission electron microscopy (TEM). Figure 3 displays TEM images of AlN nanorods mechanically removed from the wafer and deposited on a carbon grid. Compared to SEM observations, TEM measurements reveal a 5 to 10 nm diameter increase from the top to the bottom of the nanorods, resulting in very slight tapering with angles ranging from 0.08 to 0.16°, thus validating the high degree of verticality of the nanorod sidewalls. Additionally, the TEM images show the degree to which the nanorods have become faceted through the change of contrast from the centre to each edge. As observations were performed along the [$10\bar{1}0$] zone axis, this difference in contrast can be ascribed to the observation of non-polar $10\bar{1}0$ *m*-plane facets so that the AlN nanorods after the two-step etching possess a hexagonal shape. Nanorod faceting via KOH-based wet etching has already been reported in the literature for GaN nanorods [50], and therefore might also be expected for AlN. However, while the three *m*-plane facets appear to be well defined on the lower part of the nanorod, and are also discernable on the bottom part of the nanorod in Figure 2b, their observation becomes less clear on the top part, which could suggest incomplete faceting after the KOH step. Finally, TEM images reveal that only one threading dislocation (TD) is observed on the bottom part of the nanorod in Figure 3a, as well as on the top part of the nanorod in Figure 3b, while no TD is observed for the nanorod in Figure 3c. Therefore, the top-down etching steps have efficiently filtered the threading dislocations initially present in the AlN template. While dislocation filtering is to be expected after top-down etching [25], as it is for selective area growth [24], it is important to highlight that the size of the nanorod diameter plays a major role in the number of dislocations filtered. Figure 3d shows the number of dislocations from the template that can remain in the nanorod. This is calculated for different nanorod diameters and for different dislocation densities of the AlN template. Thus, if we consider a homogenous dislocation density of ~1–3 × 10^10^ cm^−2^ and a nanorod diameter of ~130 nm (vertical black line), one or no dislocations should be observed in the AlN nanorod, which correlates well with experimental data. Increasing the etched diameter core to higher values such as 500 nm could result in an AlN nanorod containing almost ten dislocations.

### 3.2. Morphology and Structural Quality of Bottom-Up Regrown AlN Nanorod

**Figure 4:**
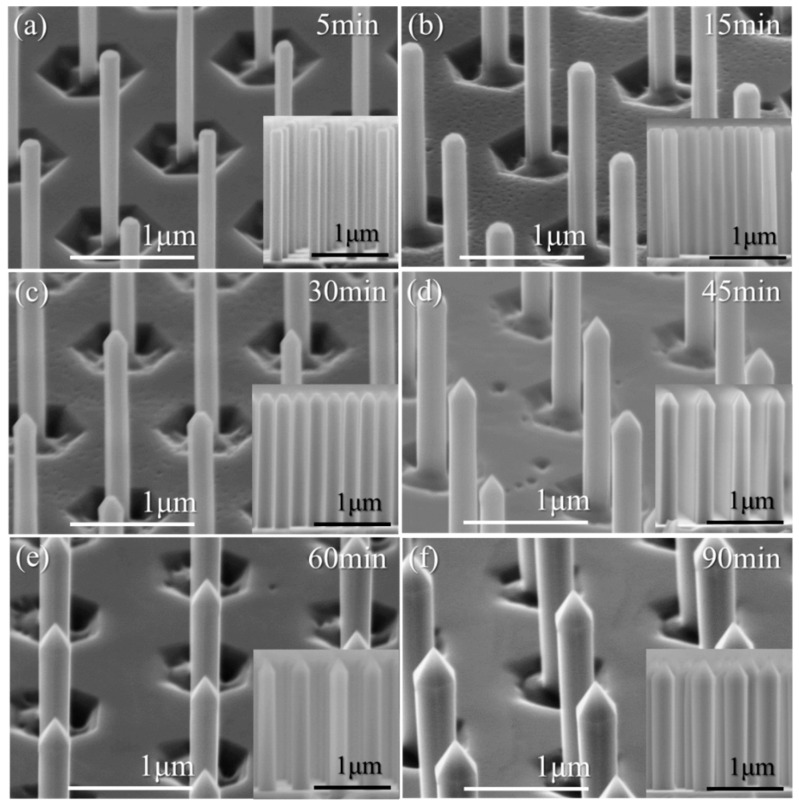
Cross-section SEM images of AlN nanorod arrays after a MOVPE regrowth time of (**a**) 5 min, (**b**) 15 min, (**c**) 30 min, (**d**) 45 min, (**e**) 60 min, and (**f**) 90 min. Each inset shows a cross-section of the AlN nanorods.

**Figure 5:**
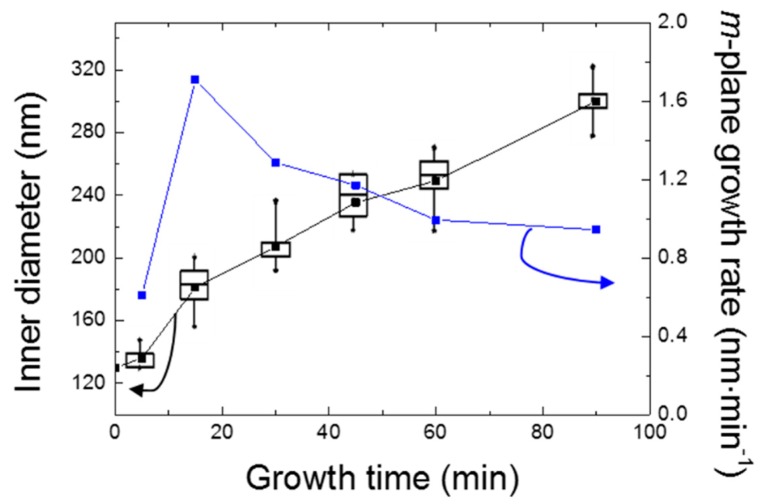
The inner diameter (in black) and m-plane growth rate (in blue) as a function of the growth time. The box plot representation displays the average (dark point in the box) and median (dark line in the box) values, the dispersion of the measured values through the interquartile deviation (black box), and the largest and smallest values (extremities of the vertical line). Note, the outer circle of a hexagon intersects each corner, while the inner circle touches the middle of each facet, such that $d_{i}=d_{o}.\sqrt{3}/2$. The *m*-plane growth rate was then obtained by extracting the increase of the inner radius of the regrown nanorod compared to the etched nanorod, i.e., $growth\;rate=(r_{i}^{MOVPE}-r_{i}^{etch})/growth\;time$.

The AlN nanorod scaffold was subsequently placed in a MOVPE reactor for the regrowth of AlN facets. Figure 4 presents SEM images of the resulting AlN nanorods for various AlN growth times, while Figure 5 displays the inner diameter (extracted from plan-view SEM images) and the *m*-plane growth rate as a function of the growth time.

The morphology of the nanorod evolves as a function of the growth time. In the early stages of growth, *m*-plane facets are visible on the nanorod sidewalls, but there is a small gap between neighbouring *m*-plane facets on the lower part. The top comprises a truncated pyramid with six ($10\bar{1}1$) semi-polar planes and a large top *c*-plane (Figure 4a,b). For intermediate growth times (Figure 4c,d), the sidewalls are solely comprised of *m*-plane facets with a truncated pyramid at the top, having a small top *c*-plane. For the highest growth times (Figure 4e,f), the nanorod still has well-defined *m*-plane sidewall facets, but the *c*-plane at the tip has been extinguished, leaving a complete pyramid on top.

Cross-section images, shown as insets in Figure 4, reveal the extremely good height uniformity resulting from the hybrid top-down bottom-up approach employed in this work. In addition, it can be observed that the verticality of the nanorod sidewalls remains high until the longest growth time is reached (Figure 4f), in which the diameter becomes larger on the top part of the nanorod compared to the bottom. As the *c*-plane is extinguished after 60 min and the top pyramid is completely formed, species on the slow-growing semi-polar planes can now only diffuse down the nanorod to the junction with the *m*-plane, thus resulting in a higher diameter at the top of the nanorods.

An increase of diameter as a function of growth time is observed from the SEM images in Figure 4 and the graph in Figure 5. The increase is more significant in the early stages of growth (<15 min), after which the increase drops and becomes linear with growth time. The transition between higher and lower growth rate at around 15 min correlates with the complete coverage of the nanorod sidewalls with *m*-plane facets. For the growth conditions employed, the AlN *m*-plane growth rate is found to be significantly lower than the *c*-plane growth rate of conventional planar AlN layers. An increase of the growth rate from ~0.6 to ~1.7 nm·min^−1^ is first observed between 5 and 15 min, which then gradually decreases to stabilize at ~1 nm·min^−1^ after ~60 min. The low value extracted after 5 min could be caused by an incubation or nucleation time. Once this time passed, growth takes place on the *m*-plane and facets start to form. For the highest growth times, the nanorod diameter is observed to be lower on the bottom. Therefore, the *m*-plane growth rate extracted from plan-view SEM pictures (i.e., top diameter) represents the maximum value.

From these observations, the AlN regrowth time to create complete sidewall facets should be chosen with care if one wants to overgrow a quantum well, since a short growth time leads to incomplete faceting, whereas a long growth time induces preferential growth on the upper part of the *m*-plane facets. For the nanorod geometry and growth conditions presented in this paper, an initial AlN growth time of 30 to 45 min would be suitable for subsequent quantum well growth.

**Figure 6:**
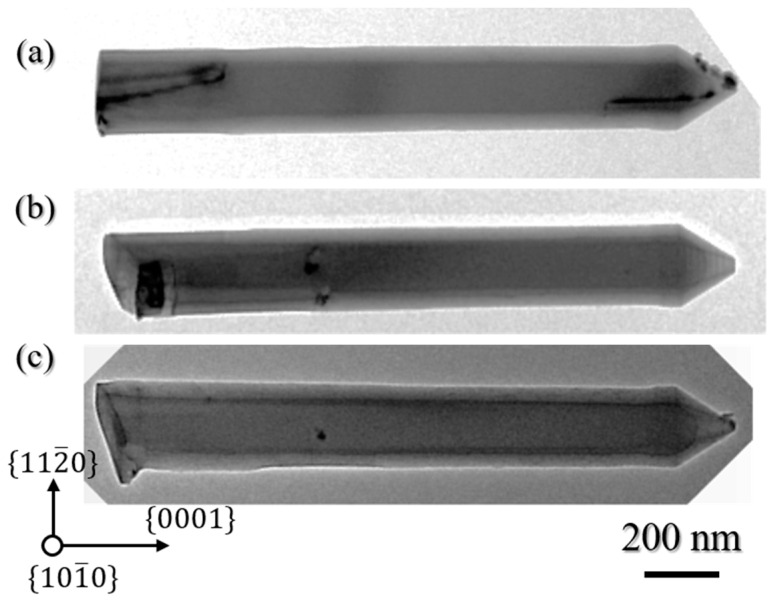
(**a**–**c**) TEM images of three different AlN faceted nanorods acquired along the [$10\bar{1}0$ zone axis. The left and right hand-side of each image corresponds to the bottom and the top of the nanorod, respectively.

Figure 6 shows TEM images of AlN faceted nanorods after 45 min growth. TEM measurements confirm the high verticality of the sidewalls with almost no fluctuation in the nanorod diameter across its height. Threading defects and pits are observed in Figure 6a–c, respectively. We ascribe these defects to TDs initially present in the AlN etched core that bend towards the lateral free surfaces and emerge on the sidewalls as pits.

### 3.3. Impact of the Etched Nanorod Morphology and Dimensions on the AlN Faceting

**Figure 7:**
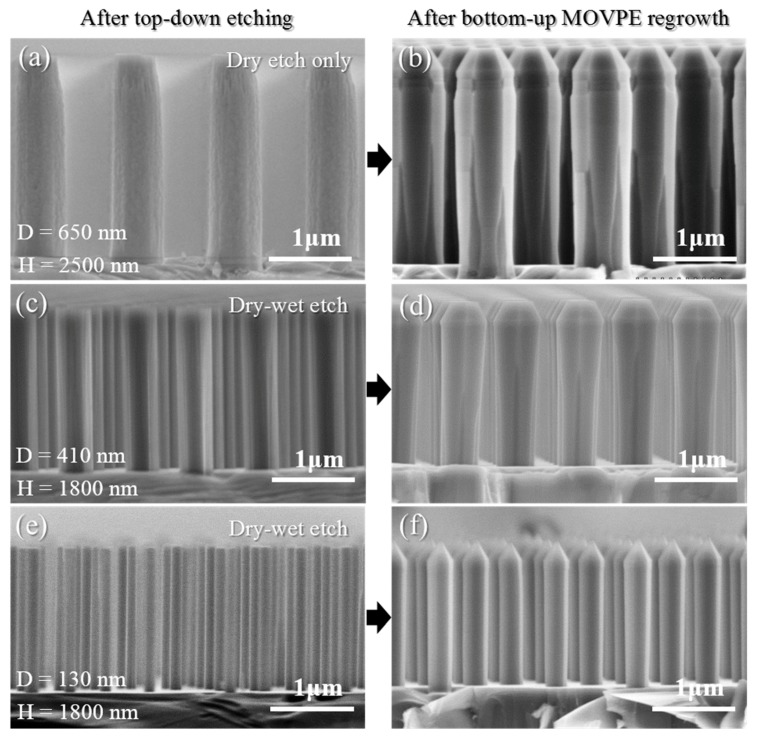
AlN etched nanorods achieved with (**a**) a 650-nm-diameter metal mask and a single step dry etch, (**b**) a 650-nm-diameter metal mask and a two-step dry-wet etch, and (**c**) a 250-nm-diameter metal mask and a two-step dry-wet etch; (**b**,**d**,**f**) 90 min AlN faceting regrowth from (**a**,**c**,**e**).

Figure 7 presents the effects of regrowing AlN on various etched AlN nanorod templates. In our previous work, we reported on the optimization of the dry etching conditions required to achieve straight AlN nanorods and the subsequent optimization of the MOVPE regrowth conditions to achieve straight and smooth sidewalls [48]. At this point in time, no additional KOH-based wet etch was used. In particular, we showed that the use of low pressure and a high V/III ratio enables a quasi-complete coverage of the AlN nanorods, leading to a smooth and straight sidewall profile (Figure 7b). 

However, etching striations observed on the top of the nanorods after dry etching (Figure 7a) induced the formation of steps on the top part of the nanorods after regrowth (Figure 7b). In addition, the junction between neighboring *m*-plane facets was not completely formed on the bottom part of the nanorods (Figure 7b). By adding the wet-etch step and employing a two-step dry-wet etching as presented in Figure 7c, the nanorod profile becomes straighter, and the etching striations can be suppressed, resulting in the absence of regrowth steps on the top of the nanorod (Figure 7d). The aim is to achieve m-plane facets along the entire side walls of the nanorods in order to achieve homogenous emission properties from any active region subsequently grown. This is aided by the reduction of the number of types of facet present in the structure in order to reduce competition in adatom incorporation on different facets. Whilst adding the wet-etch step has improved the nanorod homogeneity nearer the top of the nanorod, for initial nanorod diameters around 400 nm, the SEM images from the regrowth still show dark contrast between the *m*-planes nearer the bottom. We ascribe this to gap between the *m*-planes rather than the existence of *a*-plane facets.

In the present work, ~130 nm diameter AlN nanorods were achieved (Figure 7e) thanks to the optimization of the fabrication process, i.e., DTL exposure and lift-off, and the two-step dry-wet etching, resulting in a complete facet recovery of the AlN nanorods (Figure 7f). Therefore, complete AlN facet recovery of smooth and straight sidewalls is achieved by a careful optimization of the initial nanorod diameter along with optimum growth conditions. However, it should be pointed out that the pitch of the nanorod array can also play a role in the facet recovery mechanism. In our case, a 1.5 μm pitch was employed to allow delivery of gas reactants to the bottom part of the nanorods. One could expect that for lower pitch (<500 nm), the larger filling factor would lead to preferential growth on the top part of the nanorod and to the formation of close-packed hexagonal nanopyramids having non-straight sidewalls underneath [47].

### 3.4. Optical Properties

**Figure 8:**
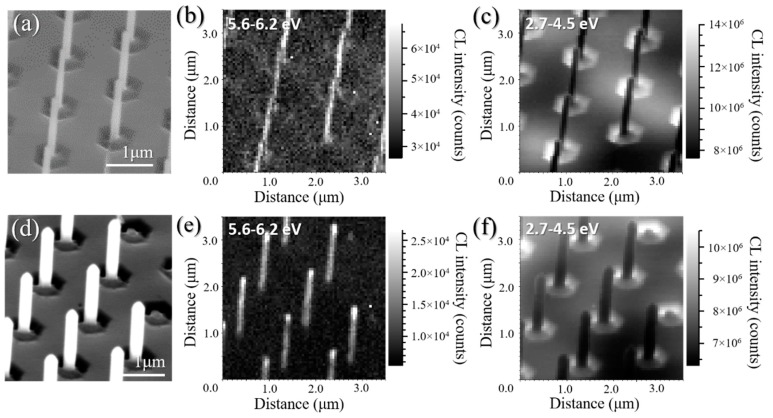
(**a**) Tilted SEM image of the AlN nanorod array after the two-step dry-wet etching process and related CL intensity map extracted between (**b**) 5.6–6.2 eV for the AlN NBE and (**c**) 2.37–4.5 eV for the defect band; (**d**) Tilted SEM image of the AlN nanorod array after 45 min AlN facet recovery and related CL intensity map extracted between (**e**) 5.6–6.2 eV for the AlN NBE and (**f**) 2.37–4.5 eV for the defect band.

The optical emission of the AlN nanorods before and after regrowth was assessed by room temperature, high-resolution cathodoluminescence (CL) hyperspectral imaging. In the top row of Figure 8, Figure 8b,c displays the AlN near band edge (NBE) and defect-related intensity maps obtained after the two-step etching, alongside the corresponding SEM image in Figure 8a. In the bottom row, Figure 8d–f shows the corresponding SEM and CL maps after 45 min AlN regrowth. The defect-related emission already present in the initial AlN template could be due to O-complexes and/or Si-complexes and other native defects, such as vacancies [51,52]. Strong NBE emission between 5.6–6.2 eV is observed from both samples, though after the overgrowth we can only extract NBE emission from the core of the nanorod, even when using lower acceleration energies. Whilst the measurement conditions have changed slightly from sample to sample, we can nevertheless deduce that the relative intensity of the NBE emission has dropped slightly for the regrown sample. This is most likely caused by an increase in material thickness, which reduces the amount of electrons reaching the core. We suspect the reason as to why we only observe AlN NBE emission from the core is that the regrowth introduces a number of point defects in the regrown shell, which will act as effective carrier traps. On a positive note, the emission in the defect band does not noticeably increase for the regrown sample, and the defect-related emission is lower for the AlN nanorods compared to the planar layer.

## 4. Conclusions

In conclusion, by employing a hybrid top-down/bottom-up approach, we fabricated a highly uniform and organized faceted AlN nanorod scaffold. The successful fabrication of AlN nanorods with nonpolar (*m*-plane) facets is achieved by creating smooth vertical nanorods with a narrow diameter thanks to the combination of a two-step dry-wet etching process and by employing optimized MOVPE AlN regrowth conditions. The AlN faceted nanorods exhibit high structural and reasonable optical quality. Such AlN nanorod templates will enable the mitigation of the quantum-confined Stark effect for subsequent active layers, will improve the structural quality of deep-UV core-shell structures (hence increasing the internal quantum efficiency), and will improve light extraction. Therefore, the hybrid top-down/bottom-up approach shows great promise for a novel, deep-UV core-shell architecture.

## Supplementary Materials

This publication is supported by multiple data sets, which are openly available here: https://doi.org/10.15125/BATH-00522.
